# Draft Genome Sequences of Two *Geobacillus* Species Strains, Isolated from Oil Wells and Surface Soil above Oil Pools

**DOI:** 10.1128/genomeA.01129-16

**Published:** 2016-10-20

**Authors:** Arnoldas Kaunietis, Anne de Jong, Raminta Pranckutė, Andrius Buivydas, Oscar P. Kuipers

**Affiliations:** a Molecular Genetics, University of Groningen, Groningen, The Netherlands; b Department of Microbiology and Biotechnology, Vilnius University, Vilnius, Lithuania

## Abstract

Here, we present the draft genome sequences of two *Geobacillus* species strains isolated from oil wells and surface soil above oil pools in Lithuania.

## GENOME ANNOUNCEMENT

*Geobacillus* spp. have Gram-positive, rod-shaped, and spore-forming cells. They grow aerobically or facultative anaerobically. Oxygen is the terminal electron acceptor, which is replaceable in some species by nitrate. They are obligately thermophilic. The temperature range for growth ranges between 37 and 75°C, with an optimum temperature of 55 to 65°C (1). *Geobacillus* species strains may be a source of novel bacteriocins or bacteriocin-like substances (BLIS) (2–4). This genus of thermophilic bacteria is also a source of enzymes for biocatalysis, and thus it has potency of application in biofuel production and other fields of biotechnology (5).

In this work, we used *Geobacillus* species strains 8 and 15 from the culture collection of the Department of Microbiology and Biotechnology of Vilnius University (Vilnius, Lithuania). These strains were isolated from Lithuanian oil wells (strain 15) and surface soil above the oil pools in Lithuania (strain 8). Both are Gram-positive, spore-forming, and rod-shaped bacteria (6). The strains were grown in nutrient broth (NB) medium at 55°C and 200 rpm. One liter of NB medium contained 10 g of tryptone, 5 g of beef extract, and 5 g of NaCl. Agar was added (1.5% [wt/vol]) for solid NB medium preparation. Cultures were inoculated to the liquid NB medium from single colonies of the solid NB medium plates. During exponential growth of the cultures, cells were collected, and genomic DNA was extracted using GenElute bacterial genomic DNA kit (Sigma-Aldrich).

The isolated DNA was sheared to 500-bp fragments in the Covaris ultrasonic device (KBioscience) for preparing the next-generation sequencing (NGS) libraries using the paired-end NEB NextGen library preparation kit. The libraries were 250-base paired-end sequenced on an Illumina HiSeq 2000. Subsequently, Velvet (7) was used to perform a *de novo* paired-end assembly on each genome, resulting in the draft genome sequences. The RAST server (8) and BAGEL3 (9) were used to annotate the genomes and to identify putative bacteriocin gene clusters, respectively.

### Accession number(s).

The genome sequences of *Geobacillus* species strains 8 and 15 have been deposited in GenBank under the accession numbers LVHY00000000 and LVHZ00000000, respectively.
